# The PERISCOPE Cohort: A Retrospective Study of Clinicopathological and TRAF7 Genetic Findings in Intraneural Perineurioma

**DOI:** 10.1111/ene.70519

**Published:** 2026-02-09

**Authors:** Eduardo Boiteux Uchôa Cavalcanti, Alessandra de La Rocque Ferreira, Nilo Sakai Júnior, Francineide Sadala de Souza, Heveline Becker de Moura, Eni Braga Da Silveira, Graciela Maria Barbosa Lacerda Martins, Marcio de Mendonça Cardoso

**Affiliations:** ^1^ Department of Neurology Rede SARAH de Hospitais de Reabilitação Brasília Distrito Federal Brazil; ^2^ Molecular Pathology Laboratory Rede SARAH de Hospitais de Reabilitação Brasília Distrito Federal Brazil; ^3^ Department of Surgical Pathology Rede SARAH de Hospitais de Reabilitação Brasília Distrito Federal Brazil; ^4^ Department of Radiology Rede SARAH de Hospitais de Reabilitação Brasília Distrito Federal Brazil; ^5^ Department of Neurosurgery Rede SARAH de Hospitais de Reabilitação Brasília Distrito Federal Brazil

**Keywords:** intraneural perineurioma, peripheral nerve sheath tumour, target nerve biopsy, tendon transfer, *TRAF7*

## Abstract

**Background:**

Intraneural perineurioma (INP) is a rare, benign peripheral nerve sheath tumour that typically presents in adolescence or early adulthood as a slowly progressive, motor‐predominant mononeuropathy or plexopathy. Although its clinicoradiological and histopathological features are well characterised, the genetic basis remains incompletely defined.

**Methods:**

We retrospectively analysed 10 patients with histologically confirmed INP diagnosed between February 2015 and December 2024. Demographic and clinical data, MRI/MR neurography findings and histopathology (immunohistochemistry and electron microscopy) were analysed. Targeted Sanger sequencing of TRAF7 exons 17–18 (WD40 domain) was performed. Interphase FISH with an EWSR1 (22q12) probe was performed on archival FFPE tissue in a subset.

**Results:**

All patients exhibited progressive motor deficits, with at least one muscle group graded ≤ 2 on the MRC scale. Sensory symptoms were present in 8/10 and pain in 4/10. MRI demonstrated fusiform nerve enlargement and homogeneous gadolinium enhancement in all cases, with T2 hyperintensity in 9/10. A pathogenic *TRAF7* p.His521Arg variant was identified in 2/9 evaluable tumours (22.2%). Tendon transfer was performed in 7/10 patients as a reconstructive strategy to improve motor function, resulting in heterogeneous functional outcomes.

**Interpretation:**

The MRI triad of fusiform enlargement, T2 hyperintensity and homogeneous enhancement strongly supports INP diagnosis and may obviate biopsy in typical cases. Our hotspot‐limited assay detected *TRAF7* mutations in only 22.2%, underscoring methodological limitations and probable genetic heterogeneity. Despite an indolent imaging appearance, INP frequently causes severe functional impairment requiring reconstructive surgery. Early recognition, structured functional monitoring and risk‐adapted intervention are essential to optimise outcomes.

## 1 Introduction

Intraneural perineurioma (INP) is a rare, benign peripheral nerve–sheath tumour characterised by a proliferation of perineurial cells within a peripheral nerve or plexus [1, 2, 3]. First described in 1964 by Da Gama et al. as ‘interstitial hypertrophic neuritis’, it is now recognised as a distinct clinicopathological entity [1, 4]. Histopathologically, INP exhibits concentric perineurial cell whorls (pseudo–onion bulb formations), with epithelial membrane antigen (EMA) immunopositivity and relative S100 protein negativity—features that distinguish it from Schwann cell–derived neoplasms [2, 3, 5, 6].

Clinically, INP typically manifests during adolescence or early adulthood as a slowly progressive mononeuropathy or plexopathy with predominant motor involvement [2, 3, 6, 7]. While early descriptions emphasised motor impairment, subsequent case series indicate that sensory disturbances and pain are more frequent than previously recognised [2, 3, 8, 9]. Diagnosis is frequently delayed owing to overlap with hereditary neuropathy with liability to pressure palsies, leprosy, multifocal motor neuropathy and entrapment syndromes [7, 10, 11, 12].

Magnetic resonance imaging (MRI), often when combined with magnetic resonance neurography, has transformed the non‐invasive diagnosis of INP. A characteristic triad—fusiform nerve enlargement, T2‐weighted hyperintensity and homogeneous gadolinium enhancement—has become a diagnostic hallmark [6, 7, 12, 13]. When concordant with clinical presentation, this pattern provides robust diagnostic support and, as several studies suggest, may obviate the need for histopathological confirmation, reducing the role of invasive biopsy in typical cases [7, 13].

From a genetic perspective, systematic investigations remain limited. The largest study to date identified *TRAF7* mutations in approximately 60% of INPs, all localised to the WD40 domain [14]. These variants are thought to promote tumorigenesis through dysregulation of the nuclear factor kappa B (NF‐κB) signalling pathway, a mechanism also implicated in meningiomas harbouring homologous variants [15]. Beyond neoplasia, *TRAF7* mutations have been associated with developmental disorders such as CAFDADD and with a spectrum of craniofacial anomalies [16, 17, 18]. Additional genetic alterations, such as 22q deletions involving *NF2* and *SMARCB1*, have been reported, although their role in INP pathogenesis remains uncertain [7, 19, 20]. While the clinical utility of genetic profiling has not yet been established, it holds promise for refining molecular characterisation, strengthening diagnostic certainty in atypical cases, and ultimately informing future risk stratification and therapeutic development [7, 14].

Despite these advances, differentiation from other peripheral nerve sheath tumours, particularly hybrid lesions, may still necessitate tissue sampling in atypical cases [7, 13, 21, 22]. Furthermore, management strategies remain controversial due to the tumour's benign nature and typically indolent course. In most cases, conservative treatment comprising ongoing clinical observation and physiotherapy is generally favored [2, 3, 6, 7]. Surgical intervention is generally reserved for progressive or function‐limiting deficits [2, 6, 7]. Owing to the intraneural growth pattern, resection is rarely feasible without risking further neurological compromise, and reconstructive procedures such as nerve grafting are often required [6, 7, 9, 23].

In this single‐centre retrospective study of 10 histologically confirmed INP cases from a Brazilian cohort, we integrated clinical, radiological, histopathological and genetic data. By evaluating the diagnostic performance of the MRI triad alongside the yield of hotspot‐targeted *TRAF7* sequencing, we provide new insights into the molecular heterogeneity of this tumour and highlight practical challenges of molecular confirmation in an era when biopsy is increasingly avoided. These findings refine current diagnostic criteria and offer perspectives for future diagnostic and therapeutic approaches.

## 2 Methods

### 2.1 Study Design and Ethics

This retrospective observational cohort study, designated as the PERISCOPE study (Perineurioma Study Cohort: Phenotype Exploration), included patients diagnosed with INP between February 2015 and December 2024 at a Brazilian tertiary referral centre for peripheral nerve disorders. The study followed the Strengthening the Reporting of Observational Studies in Epidemiology (STROBE) guidelines.

Ethical approval was granted by the Institutional Review Board (Comitê de Ética em Pesquisa) of Rede SARAH de Hospitais de Reabilitação (CAAE: 77712724.0.0000.0022). Due to the retrospective nature of the study and the use of de‐identified data, informed consent was waived.

### 2.2 Patient Selection

Eligible patients were identified from institutional neurology, neurosurgery and pathology databases. Inclusion criteria were: (1) histopathological confirmation of INP and (2) availability of complete core diagnostic elements (clinical, imaging and histopathological data). Exclusion criteria included: (1) incomplete records, (2) alternative diagnoses (e.g., schwannomas, neurofibromas, hybrid nerve sheath tumours) or (3) prior interventions confounding the diagnostic process.

### 2.3 Clinical Assessment

Demographic variables (age, sex), symptom onset and duration, anatomical distribution, and presenting features were extracted from medical records. Motor strength was graded using the Medical Research Council (MRC) scale and categorised as severe (MRC ≤ 2), moderate (MRC = 3), or mild (MRC ≥ 4). Sensory symptoms and pain (including neuropathic descriptors when documented) were recorded. Disease progression was categorised as stable or progressive based on follow‐up documentation. In addition, medical procedures—including diagnostic biopsy, tendon transfer surgery and tumour resection with nerve grafting—were systematically registered.

### 2.4 Neuroimaging Assessments

All patients underwent 1.5T or 3.0T MRI following the institutional protocol for peripheral nerve evaluation, which included axial and coronal T1‐weighted, T2‐weighted and post‐gadolinium T1‐weighted sequences. MRN was selectively performed to better delineate fascicular architecture. Studies were reviewed by an experienced radiologist. The following imaging features were systematically assessed: (1) lesion morphology; (2) T2 signal intensity; (3) contrast enhancement pattern; (4) lesion extent (> 10 cm vs. ≤ 10 cm) and (5) nerve cross‐sectional area (CSA) at both pre‐lesion and lesion sites. Serial imaging was defined as two or more MRI examinations performed at least 6 months apart. Radiological progression was defined a priori as any of the following: ≥ 10% increase in maximal CSA, ≥ 10 mm increase in longitudinal extent, or new fascicular involvement/enhancement.

### 2.5 Histopathological, Immunohistochemical and Ultrastructural Analysis

Peripheral nerve specimens obtained for prior diagnostic purposes were independently re‐evaluated by two neuropathologists. Entries marked as ‘not available’ in the tables indicate either archival slides unsuitable for further analysis or incomplete historical laboratory records.

Formalin‐fixed, paraffin‐embedded (FFPE) tissue sections were stained with haematoxylin and eosin (H&E) and examined via light microscopy. Immunohistochemistry was performed on FFPE sections using antibodies against EMA, S100 protein, claudin‐1 and neurofilament protein (NFP). Staining was semiquantitatively assessed for intensity (strong, weak, negative) and distribution (diffuse, focal, inconclusive) with internal and external controls. Diagnostic confirmation required pseudo‐onion bulb formations with EMA‐positive/S100‐negative perineurial cell whorls surrounding axons.

Ultrastructural analysis was performed in eight patients. Biopsies were fixed in glutaraldehyde, post‐fixed in osmium tetroxide, and embedded in epoxy resin. Ultrathin sections were stained with uranyl acetate and lead citrate and examined under transmission electron microscopy for characteristic perineurial features, including concentric perineurial cell lamellae and discontinuous basal lamina.

### 2.6 Genetic Analysis

Genomic DNA was extracted from FFPE tissue using standard protocols. Targeted Sanger sequencing was performed on exons 17 and 18 of the *TRAF7* gene (WD40 domain). Sequence data were aligned to the GRCh38 human reference genome. Variant calling and interpretation followed the American College of Medical Genetics and Genomics (ACMG) guidelines [24]. All variants were cross‐checked against the ClinVar and gnomAD databases for population frequency and pathogenicity classification.

Interphase fluorescence in situ hybridisation (FISH) was performed in a subset of five patients, limited to those with available FFPE tumour tissue, to assess abnormalities of chromosome 22. Four‐micron sections were hybridised with locus‐specific probes, including LSI *EWSR1* (22q12) (Abbott/Vysis) according to the manufacturer's protocol. At least 100 consecutive, non‐overlapping nuclei were scored using a Zeiss Axio Imager Z1 fluorescence microscope equipped with FISHView software (Applied Spectral Imaging, Migdal HaEmek, Israel). Nuclei were considered positive for deletion/monosomy when ≥ 10% demonstrated loss of one *EWSR1* signal.

Whole‐exome sequencing (WES) and whole‐genome sequencing (WGS) were unavailable due to resource constraints.

### 2.7 Statistical Analysis

Descriptive statistics were used to summarise clinical, radiological, pathological and molecular data. Categorical variables were reported as frequencies and percentages; continuous variables were summarised using means, medians and ranges, as appropriate. Data were analysed using Microsoft Excel (version 2024).

## 3 Results

### 3.1 Demographic and Clinical Characteristics

Ten patients (six females, four males) with histologically confirmed INP were included (Table 1). Median age at first evaluation at our centre was 26.5 years (interquartile range [IQR]: 23–41; mean: 30.4 years; range: 13–48), and the median age at symptom onset was 21 years (IQR: 14–27; mean: 22 years; range: 9–37). Affected nerves included the common fibular (*n* = 3), ulnar (*n* = 2), sciatic (*n* = 2), lumbosacral plexus (*n* = 1), median (*n* = 1) and radial (*n* = 1).

**TABLE 1:** Demographic and clinical features of patients with intraneural perineurioma.

Patient ID	Sex	Age at first evaluation (years)	Age at symptom onset (years)	First presenting symptom	Affected nerve	Laterality
P1	Female	25	16	Foot drop (dorsiflexion weakness)	Sciatic nerve (common fibular nerve + tibial nerve)	Left
P2	Male	22	14	Foot drop (dorsiflexion weakness)	Sciatic nerve	Left
P3	Female	47	37	Thenar muscle atrophy	Median nerve	Right
P4	Female	34	27	Wrist and finger extension weakness	Radial nerve	Right
P5	Female	48	24	Anterior leg pain + foot drop	Lumbosacral plexus + sciatic nerve	Right
P6	Male	41	37	Foot drop (dorsiflexion weakness)	Common fibular nerve	Right
P7	Female	23	14	First dorsal interosseous muscle atrophy	Ulnar nerve	Left
P8	Male	28	23	Foot drop (dorsiflexion weakness)	Common fibular nerve	Left
P9	Male	23	19	Intrinsic hand muscle atrophy (hypothenar)	Ulnar nerve	Right
P10	Female	13	9	Foot drop (dorsiflexion weakness)	Common fibular nerve	Right

All patients presented with progressive motor deficits, and each exhibited at least one muscle group with severe weakness (MRC ≤ 2). Sensory symptoms, typically mild‐to‐moderate hypoesthesia, were present in 8/10 patients (80%). Pain was reported in 4/10 patients (40%), with neuropathic features noted in two cases (P5, P8) and activity‐induced myofascial pain in two others (P7, P10). None of the patients fulfilled clinical criteria for neurofibromatosis types 1 or 2.

### 3.2 Neuroimaging Findings

**FIGURE 1:**
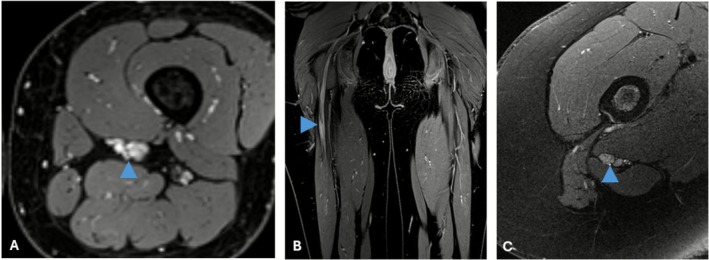
MRI findings in patients with intraneural perineurioma. (A) Axial VIBE fat‐suppressed post‐contrast image (Patient 1) showing fusiform nerve enlargement with homogeneous contrast enhancement (arrowhead). (B) Coronal VIBE fat‐suppressed post‐contrast sequence (Patient 10) demonstrating longitudinal extension and homogeneous enhancement. (C) Axial T2‐weighted fat‐suppressed image (Patient 10) depicting hyperintense signal and altered fascicular architecture (arrowhead).

MRI findings were available for all patients (Table 2; Figure 1). Fusiform nerve enlargement was observed in 10/10 of cases. T2‐weighted hyperintensity was present in 90% (9/10), and homogeneous gadolinium enhancement was observed in all patients (100%). Lesions were uniformly isointense on T1‐weighted images.

**TABLE 2:** Neuroimaging findings in patients with intraneural perineurioma.

Patient ID	P1	P2	P3	P4	P5	P6	P7	P8	P9	P10
Affected nerve	Sciatic nerve (common fibular nerve + tibial nerve)	Sciatic (fibular nerve)	Median	Radial	Lumbosacral plexus + sciatic	Common fibular nerve	Ulnar	Common fibular nerve	Ulnar	Common fibular nerve
Nerve thickening type	Fusiform	Fusiform	Fusiform	Fusiform	Fusiform	Fusiform	Fusiform	Fusiform	Fusiform	Fusiform
Maximum lesion extension > 10 cm	Yes (fibular: 12 cm, tibial: no, 7.1 cm)	Yes (sciatic: 11 cm, fibular: 12 cm)	No (5.8 cm)	No (9.0 cm)	Yes (45.0 cm)	No (2.6 and 1.0 cm)	No (4.0 cm)	No (8.0 cm)	No (6.5 cm)	Yes (18 cm)
Pre‐lesion nerve CSA (1 cm proximal to lesion)	8.3 mm^2^/12.0 mm^2^	59 mm^2^/8.0 mm^2^	5.8 mm^2^	Not reported	12.0 mm^2^	9.0 mm^2^	Not reported	6.0 mm^2^	15.0 mm^2^	Not reported
Maximum CSA at lesion site	33 mm^2^/34 mm^2^	82 mm^2^/not reported	44.0 mm^2^	23.0 mm^2^	97.0 mm^2^	70.0 mm^2^	43.0 mm^2^	44.0 mm^2^	40.0 mm^2^	42.0 mm^2^
Lesion involves entire nerve area	No (both)	No (both)	Yes	Yes	Not reported	Yes	Yes	Yes	Yes	Yes
T1 isointense signal	Yes	Yes	Yes	Yes	Yes	Yes	Yes	Yes	Yes	Yes
T2 hyperintense signal	Yes	Yes	Yes	Yes	No	Yes	Yes	Yes	Yes	Yes
Contrast enhancement	Yes	Yes	Yes	Yes	Yes	Yes	Yes	Yes	Yes	Yes
Growth over time (yes/no/NSA)	No (both)	NSA	NSA	No	No	NSA^a^	No	No	NSA	No
Time interval between exams	3 years	Not reported	Not reported	3 years	5 years	18 years	7 years	1 year	Not reported	7 months

Abbreviations: CSA, cross‐sectional area; NSA, no serial assessment.

^a^ Patient underwent excision of the lesion and a nerve graft procedure.

Lesions extended over more than 10 cm in four patients (40%) and involved the entire cross‐sectional diameter of the nerve in 7/9 cases (77.8%). Lesion CSA ranged from 23 to 97 mm [2]. Pre‐lesion CSA, available in seven patients, ranged from 5.8 to 59 mm [2]. No progression was observed on serial MRI follow‐up (7 months–7 years) in the six available cases (Table 2).

### 3.3 Histopathological, Immunohistochemical and Ultrastructural Findings

**FIGURE 2:**
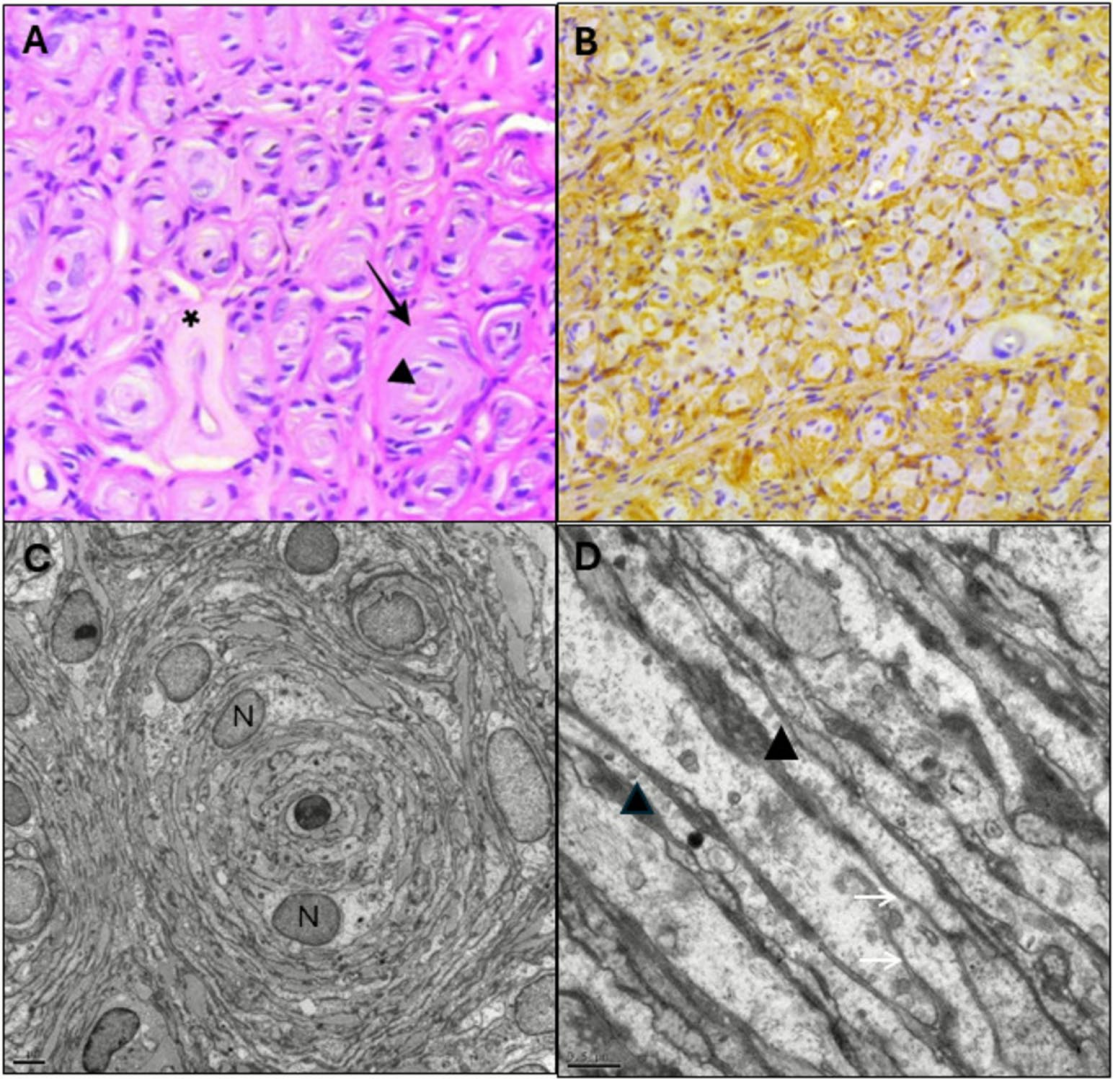
Histological, immunohistochemical, and ultrastructural features of intraneural perineurioma. (A) Haematoxylin and eosin staining (×400) showing concentric whorls of perineurial cells encasing central axons (arrowheads); hyalinized vessel indicated (asterisk). (B) EMA immunostaining demonstrates strong membranous positivity in pseudo‐onion bulb structures (×400). (C) High‐power image showing elongated perineurial cells and pseudo‐onion bulb architecture; nuclei marked (N); scale bar: 4 μm. (D) Electron microscopy revealing interdigitating cytoplasmic processes with pinocytotic vesicles (black arrows) and discontinuous basal lamina (white arrows); scale bar: 0.5 μm.

All cases demonstrated the histological hallmarks of INP, including pseudo‐onion bulb formations consisting of concentric perineurial cell whorls (Table S1; Figure 2). EMA staining was strongly positive in all samples. S‐100 protein staining was focally positive in three patients (30%), consistent with residual Schwann cell components. Claudin‐1, a marker of perineurial differentiation, was positive in five cases (P2, P7, P8, P9, P10); data were unavailable in others due to archival limitations. Neurofilament protein (NFP) positivity was observed in five patients (50%), reflecting variable axonal preservation.

Electron microscopy, performed in eight cases, revealed consistent ultrastructural features, including interdigitating perineurial processes, pinocytotic vesicles and discontinuous basal lamina, confirming the ultrastructural features of INP (Table S1; Figure 2).

### 3.4 Genetic Findings

Targeted Sanger sequencing of *TRAF7* exons 17 and 18 was successfully performed in 9/10 patients. One patient (P5) was excluded due to poor DNA sample quality for analysis. A pathogenic heterozygous missense variant, c.1562A>G (p.His521Arg), was detected in two patients (P1, P8). No additional pathogenic or likely pathogenic *TRAF7* variants were identified.

Interphase FISH was performed in 5/10 cases using a locus‐specific *EWSR1* probe (22q12) as a surrogate marker for 22q status. Analysis was limited by the small amount of available FFPE tissue. Among the evaluated samples, 3/5 (P1, P2, P8) demonstrated evidence of 22q signal loss, defined by the prespecified threshold of ≥ 10% of nuclei. The remaining two cases (P3, P7) were non‐informative due to hybridisation failure. These findings should be interpreted with caution, as the probe targets only the EWSR1 locus (22q12) and cannot detect gene‐level alterations elsewhere on 22q.

### 3.5 Treatment and Outcomes

The median interval from symptom onset to first intervention was 34.5 months (IQR: 27–50; mean: 48.1 months, range: 12–173). Diagnostic targeted fascicular biopsy was performed in nine patients without postoperative neurological worsening, supporting the safety of the procedure when histological confirmation is required.

Three patients (P1, P5, P7) were managed conservatively with physiotherapy and remained clinically stable over follow‐up durations ranging from 34 to 85 months (Table S2). One patient (P6) underwent tumour resection followed by sural nerve grafting and tendon transfer. The remaining six patients underwent tendon transfer procedures for functional improvement.

Tendon transfer was performed in seven patients (P2, P3, P4, P6, P8, P9, P10), targeting restoration of dorsiflexion in cases of common fibular nerve involvement or intrinsic hand function in cases of median or ulnar nerve lesions (Table S2). One patient (P8) remained in active rehabilitation at the time of reporting.

## 4 Discussion

This retrospective cohort expands current understanding of INP by integrating clinical, radiological, histopathological and genetic data from 10 histologically confirmed cases. Our findings corroborate established diagnostic hallmarks, while drawing attention to underrecognised clinical features, the limited yield of targeted TRAF7 sequencing, and practical implications for management and functional outcomes.

### 4.1 Clinical, Radiological and Histopathological Insights

Consistent with previous series, INP predominantly affected adolescents and young adults, with all patients presenting a slowly progressive, motor‐predominant neuropathy [2, 3, 8, 9]. Importantly, every subject included at least one muscle group with severe weakness (MRC ≤ 2), underscoring the potential for substantial motor impairment despite the tumour's benign histopathology. Pain and sensory symptoms, often underemphasised in earlier literature, were reported in a considerable proportion of our cohort, aligning with more recent observations that nociceptive or dysaesthetic features may be more common than previously appreciated [3, 8, 9]. The pathophysiology of pain in INP is not fully elucidated, but potential mechanisms include perineurial inflammation, fascicular disruption and blood–nerve barrier dysfunction [25, 26].

MRI findings were consistent across all patients, with fusiform nerve enlargement, T2 hyperintensity and homogeneous gadolinium enhancement, features well‐established in the diagnosis of INP [2, 7, 12, 13]. These imaging characteristics reinforce the growing consensus that, in patients with classical clinical and radiological features, MRI may be sufficient to establish the diagnosis, obviating the need for confirmatory nerve biopsy and the associated risk of fascicular injury [3, 13]. Lesions exceeded 10 cm in 40% and encompassed entire cross‐sections in 77.8%, illustrating the diffuse nature of INP, which may limit surgical options. These results support an imaging‐first diagnostic pathway, with biopsy reserved for atypical presentations.

Histologically, all cases exhibited pseudo‐onion bulb formations and strong EMA immunoreactivity, hallmark features of INP. This diagnostic fingerprint has remained consistent across all major histopathological series [2, 3, 7, 10]. The presence of focal S‐100 positivity in 30% likely reflects residual Schwannian elements rather than diagnostic ambiguity. Claudin‐1 and neurofilament staining further supported perineurial origin and variable axonal preservation.

### 4.2 Genetic Findings and Implications

Pathogenic *TRAF7* variants were identified in two of nine successfully sequenced tumours (22.2%), a yield lower than the approximately 60% reported in the largest prior series [14]. Several factors may explain the lower detection rate of *TRAF7* mutations. First, hotspot‐limited Sanger sequencing was restricted to exons 17 and 18, where all previously reported variants clustered [14]. Second, DNA quality from FFPE tissue may have constrained assay sensitivity. Third, population heterogeneity could contribute to differing variant frequencies.

Interphase FISH detected 22q abnormalities in three of five evaluable cases, in line with earlier reports, but its utility was limited by incomplete probe coverage and occasional hybridisation failure [27]. In clinical practice, FISH may serve as a cost‐effective screening tool when tissue is available; however, comprehensive genomic characterisation is more reliably achieved with next‐generation sequencing [14]. Whole‐exome or genome‐wide approaches offer broader coverage, although these techniques were not available in our setting at the time most biopsies were obtained.

At present, routine genetic testing neither substitutes for clinicoradiological diagnosis nor permits prognostic stratification; instead, it underscores the need for broader molecular profiling to identify alternative pathogenic mechanisms and refine genotype–phenotype correlations.

### 4.3 Management and Outcomes

Management strategies remain challenging. In this cohort, most patients developed severe motor deficits with substantial functional impairment, ultimately requiring surgical intervention. Tendon transfer procedures were performed in most cases, a rate notably higher than in previous series where conservative management prevailed [3, 6, 7].

The severe loss of motor function, evidenced by at least one muscle group with severe weakness (MRC ≤ 2) in all patients, suggests that despite the tumour's radiological indolent appearance, progressive axonal degeneration may occur silently, leading to irreversible muscle denervation. The reliance on tendon transfer likely reflects the chronicity and extent of axonal loss by the time of diagnosis. These findings question the adequacy of observation alone and underscore the need for early recognition and risk stratification to prevent irreversible motor loss.

Outcomes following tendon transfer were heterogeneous, reflecting the complexity of restoring function after chronic denervation [6, 7]. The absence of standardised outcome measures limits direct comparison across cases and precludes generalisation. This variability reinforces the importance of individualised surgical planning, realistic prognostic counselling, and validated functional assessments. Tendon transfer may improve the quality of life in selected patients, but expectations should remain cautious.

Surgical resection with nerve grafting, performed in one patient with an early severe deficit, illustrates the high morbidity associated with intraneural tumour excision. As emphasised in previous studies, complete resection is technically demanding and often necessitates reconstruction [3, 6, 7, 9]. Surgery should therefore be reserved for select cases, particularly those with short‐segment involvement and rapid progression.

Taken together, these findings support a shift toward earlier and more structured management. Despite its benign histology, INP may lead to disabling motor outcomes if not recognised and addressed on time. Multidisciplinary strategies emphasising early detection, functional monitoring, and tailored interventions are essential to optimise long‐term outcomes.

### 4.4 Limitations and Future Directions

This study is limited by its retrospective design, small sample size, and single‐centre setting, which may bias the cohort toward more severe or surgically treated cases. Genetic testing was confined to exons 17 and 18 of TRAF7, thereby potentially missing mutations in other regions or in alternative genes implicated in intraneural perineurioma, such as NF2 or broader 22q abnormalities.

Future research should prioritise multicentre, prospective cohorts using standardised functional outcome measures. Advanced imaging modalities, including diffusion tensor imaging, MR neurography, or ultra–high‐field (7‐T) MRI, may further enhance diagnostic specificity. Integrating systematic pain and quality‐of‐life assessments will also be critical to better capture patient‐reported outcomes and to guide individualised therapeutic decision‐making.

## 5 Conclusions

In summary, our study reinforces the clinical and imaging hallmarks of INP, demonstrates the functional burden of motor deficits, and provides new insights into the variable yield of TRAF7 targeted genetic testing. By situating these findings within a Latin‐American cohort, we highlight the limitations of molecular diagnosis in real‐world practice. Together, these results contribute to refining diagnostic strategies and lay the groundwork for future multicentre research.

## Author Contributions

**Eduardo Boiteux Uchôa Cavalcanti:** conceptualisation, data curation, investigation, formal analysis, writing – original draft and review. **Alessandra de La Rocque Ferreira:** genetic analysis, data curation, methodology, writing – review and editing. **Nilo Sakai Júnior:** genetic analysis, data curation, methodology, writing – review and editing. **Francineide Sadala de Souza:** histopathological evaluation, data curation, writing – review and editing. **Heveline Becker de Moura:** histopathological evaluation, data curation, writing – review and editing. **Eni Braga Da Silveira:** electron microscopy resources, writing – review and editing. **Graciela Maria Barbosa Lacerda Martins:** MRI data collection, analysis and writing – review and editing. **Marcio de Mendonça Cardoso:** conceptualisation, surgical procedures, data curation, writing – review. All authors have read and approved the final version of the manuscript.

## Funding

The authors have nothing to report.

## Ethics Statement

This study was approved by the Institutional Review Board (Comitê de Ética em Pesquisa) of Rede SARAH de Hospitais de Reabilitação (CAAE: 77712724.0.0000.0022) and conducted in accordance with the Declaration of Helsinki. Due to the retrospective nature of the study and de‐identified patient data, the requirement for informed consent was waived.

## Conflicts of Interest

The authors declare no conflicts of interest.

## Supporting information


**Table S1:** ene70519‐sup‐0001‐TableS1.xlsx.

**Table S2:** ene70519‐sup‐0002‐TableS2.xlsx.

**Data S1:** ene70519‐sup‐0003‐DataS1.docx.

## Data Availability

The data supporting this study's findings are available upon reasonable request from the corresponding author, subject to institutional and ethical approvals.
