# Supplementary material for: The PERISCOPE Cohort: A Retrospective Study of Clinicopathological and TRAF7 Genetic Findings in Intraneural Perineurioma
Source: Eur J Neurol. 2026 Feb 9;33(2):e70519. doi: 10.1111/ene.70519 (PMC12884198; doi:10.1111/ene.70519)
Supplement: Supplementary file 3 — Data S1: ene70519‐sup‐0003‐DataS1.docx. [file ENE-33-e70519-s002.docx]

Unmasking a Rare Nerve Tumor: New Insights from the PERISCOPE Cohort Study

Intraneural perineurioma (INP) is a rare nerve tumor that typically affects children, teenagers, and young adults. Although considered “benign,” it can progressively damage motor nerves in the arms or legs, leading to significant weakness and disability. Because it often mimics more common nerve conditions, diagnosis is frequently delayed.

The PERISCOPE Cohort is one of the most detailed studies of INP to date. We followed 10 patients over nearly a decade, combining clinical examination, advanced MRI imaging, nerve tissue analysis, and targeted genetic testing to better understand this tumor’s behavior, diagnosis, and management.

Our key findings:

- MRI scans consistently showed characteristic features of INP, including fusiform nerve enlargement and uniform contrast enhancement. These imaging markers may allow clinicians to recognize INP earlier and, in some cases, avoid invasive biopsies.
- Microscopic analysis confirmed the diagnosis in all cases, showing “pseudo-onion bulb” formations—concentric layers of perineurial cells around nerve fibers—validated by immunohistochemistry and electron microscopy.
- Contrary to a previous report, only 2 of 9 patients had mutations in the TRAF7 gene. This suggests greater genetic diversity in INP than previously recognized and highlights the need for broader molecular studies.
- Clinically, all patients had significant motor deficits. Most required tendon transfer surgeries to regain essential functions, such as walking or hand movement. These outcomes challenge the belief that INP can always be managed conservatively.

Why is this study important?

Our findings suggest that INP, while benign in pathology, can cause major functional impairment. MRI may serve as a key non-invasive diagnostic tool. Broader genetic testing may uncover new disease mechanisms. Most importantly, early detection and timely treatment could preserve mobility and quality of life for affected individuals.

INP is rare, but its consequences are real. Improved awareness, earlier diagnosis, and a multidisciplinary approach can change the trajectory for patients.
